# Inducible and reversible RNA N^6^-methyladenosine editing

**DOI:** 10.1038/s41467-022-29665-y

**Published:** 2022-04-12

**Authors:** Huaxia Shi, Ying Xu, Na Tian, Ming Yang, Fu-Sen Liang

**Affiliations:** Department of Chemistry, Case Western Reserve University, 2080 Adelbert Road, Cleveland, OH 44106 USA

**Keywords:** Epigenomics, Methylation, CRISPR-Cas9 genome editing

## Abstract

RNA modifications, including N^6^-methyladenosine (m^6^A), have been reported to regulate fundamental RNA processes and properties, and directly linked to various human diseases. Methods enabling temporal and transcript/locus-specific editing of specific RNA modifications are essential, but still limited, to dissect the dynamic and context-dependent functions of these epigenetic modifications. Here, we develop a chemically inducible and reversible RNA m^6^A modification editing platform integrating chemically induced proximity (CIP) and CRISPR methods. We show that m^6^A editing can be temporally controlled at specific sites of individual RNA transcripts by the addition or removal of the CIP inducer, abscisic acid (ABA), in the system. By incorporating a photo-caged ABA, a light-controlled version of m^6^A editing platform can be developed. We expect that this platform and strategy can be generally applied to edit other RNA modifications in addition to m^6^A.

## Abstract (web summary)

RNA modifications, including N6-methyladenosine (m6A), have been reported to regulate fundamental RNA processes and properties, and directly linked to various human diseases. Here, the authors develop a chemically inducible and reversible RNA m6A modification editing platform integrating chemically induced proximity (CIP) and CRISPR methods.

## Introduction

Post-transcriptional RNA modifications, including N^1^-methyladenosine (m^1^A), pseudouridine (Ψ), N^7^-methylguanosine (m^7^G), N^6^-methyladenosine (m^6^A), 5-methylcytosine (m^5^C), have recently been discovered as another layer of epigenetic regulation in gene expression^1–3^. Among these RNA modifications, m^6^A is the most prevalent in eukaryotic cells and plays important role in cellular functions, RNA-protein interaction, and the development of various human diseases^4,5^. The RNA m^6^A modification is installed by the “writers” METTL3-METTL14 complex and other auxiliary proteins^6–8^, and removed by m^6^A “erasers”, AlkB homolog 5 (ALKBH5) or fat-mass and obesity-associated protein (FTO)^9,10^. In addition, RNA m^6^A “readers” including YTH protein family, eIF3, and HNRPC, recognize m^6^A and mediate RNA metabolism and functions, including splicing^11,12^, export^13^, stability^14^, and translation^15,16^.

Overexpression or knockdown/knockout of m^6^A writers/erasers/readers has been used to study the biological functions of RNA m^6^A modification. However, these methods globally change the m^6^A levels on various endogenous RNA transcripts, making it difficult to dissect the functional contribution of individual m^6^A modifications. The recent development of clustered regularly interspaced short palindromic repeats (CRISPR)-based technologies has revolutionized the biological and medical research^17–20^. Besides genome editing, fusion proteins incorporating the effectors with different activities and catalytically inactive Cas (dCas) proteins have expanded the applications of the CRISPR systems^21–23^. Methods that fuse dCas protein (e.g., dCas9, 13b or Rx) with m^6^A “writer”, “eraser” or “reader” (e.g., METTL3, METTL3-METTL14, ALKBH5, FTO, or YTHDFs) have been developed to achieve site-specific editing or recognition of RNA m^6^A modifications^24–29^. Along with customizable guide RNAs, these tools allow researchers to study the functions of individual m^6^A modifications on specific RNA transcripts and facilitate our understanding of RNA epigenetic regulation. One limitation in most of these methods is the lack of specific temporal control that is important to understand the dynamic regulatory processes of RNA modifications and their biological consequences.

**Fig. 1:**
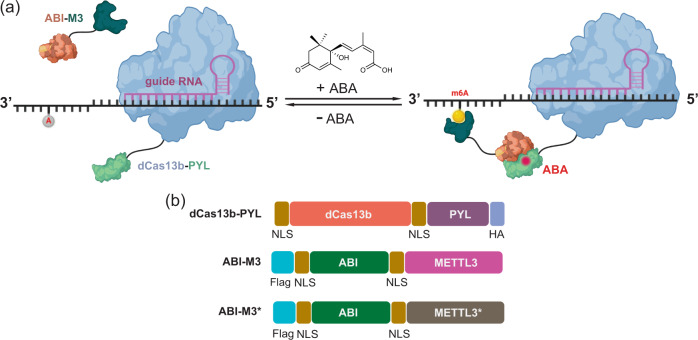
The CIP/CRISPR-based inducible and reversible m^6^A writing platform. **a** dCas13b-PYL can be localized to the target adenosine site through gRNA. The presence of ABA induces the dimerization between PYL and ABI fusion proteins, which recruits ABI-M3 to the target locus for m^6^A writing. The induced m^6^A writing can be reversed by removing ABA and thus releases ABI-M3 from the RNA site. **b** DNA plasmids constructed for the inducible m^6^A writing system, expressing fusion proteins dCas13b-PYL, ABI-M3 (wide-type METTL3), and ABI-M3* (METTL3 D395A mutant).

Herein, we developed an inducible and reversible m^6^A editing system based on CRISPR/dCas13b and abscisic acid (ABA)-based chemically induced proximity (CIP) technologies (Fig. 1a), which enables transcript/locus-specific and temporally controlled writing or erasing of RNA m^6^A modification by ligand-induced recruitment or release of the m^6^A writer or eraser proteins at the target transcript sites in cells. We showed that m^6^A writing can be achieved selectively at the targeted sites on specific RNA transcripts through conditionally recruiting METTL3. Importantly, we demonstrated that the m^6^A writing can be induced rapidly upon ABA addition and the effects can be reversed by the removal of ABA. Furthermore, by replacing the m^6^A writer METTL3 with the eraser ALKBH5, we showed that the inducible m^6^A demethylation at specific RNA transcripts was efficient. Finally, to introduce further spatiotemporal control, we incorporated a photo-caged ABA inducer and demonstrated that the inducible m^6^A writing can be successfully controlled by UV light. Taken together, we established a spatiotemporal-specific epitranscriptome editing platform for m^6^A writing and erasing on targeted RNA transcripts using a chemical ligand and light. We expect that this strategy can be readily applied to develop spatiotemporal-specific editing tools for other RNA modifications and will contribute to our understanding of RNA epigenetic regulations.

## Results

### Design of the ABA-inducible RNA m^6^A writing system

To achieve the temporal control of m^6^A editing, we integrated the CIP method with the RNA-targeting dCas13b CRISPR system to develop an inducible editing platform. In the CIP system, the inducer (e.g., ABA) triggers the association between two unique inducer-binding adaptor proteins (e.g., PYL and ABI) that are fused individually to two proteins of interest (POIs). Depending on the choice of POIs, a variety of downstream biological events can be specifically controlled by these chemical inducers^30–33^. For example, the ABA-based CIP systems have been successfully combined with the CRISPR/dCas9 or CRISPR/Cas-inspired RNA-targeting system (CIRTS) system to achieve inducible epigenome editing or RNA A-to-I base editing^34,35^. To develop an inducible m^6^A writing system, we cloned DNA constructs expressing fusion proteins of dCas13b-PYL and ABI-METTL3 (ABI-M3) (Fig. 1b). HA and FLAG tags were fused to dCas13-PYL and ABI-M3 individually for detection purposes and a nuclear localization signal (NLS) sequence was inserted in these fusion proteins to facilitate their nuclear localization, which has been shown to increase editing efficiency^25^. We anticipated that dCas13b-PYL can be targeted, through the custom guide RNA (gRNA), to the desired locus on specific RNA transcripts while ABI-M3 cannot localize on its own. Upon ABA addition, ABI-M3 will be recruited to the gRNA/dCas13b-PYL targeting site, through ABA-induced ABI-PYL heterodimerization, resulting in the increase of the m^6^A level at the targeted site on the specific RNA transcripts (Fig. 1a). We have previously demonstrated that ABA-induced dimerization and effects can be rapidly reversed by removing ABA^34,36^. We expect that ABA withdrawal will lead to the release of ABI-M3 from the RNA sites and result in a decrease in the m^6^A level. To eliminate any unwanted effects except the methyltransferase activity, we chose to include only the core catalytic domain, which was reported to be sufficient to catalyze m^6^A writing^37–39^. To confirm that the observed m^6^A level changes were caused by the recruited m^6^A-writing enzymatic activities, we also cloned a catalytic inactive version of METTL3, the ABI-M3* (D395A mutant), which lacks the m^6^A writing activity^24,25^. The constitutively active dCas13b-METTL3 fusion (dCas13b-M3) was also cloned for comparison.

### ABA-inducible m^6^A writing

**Fig. 2:**
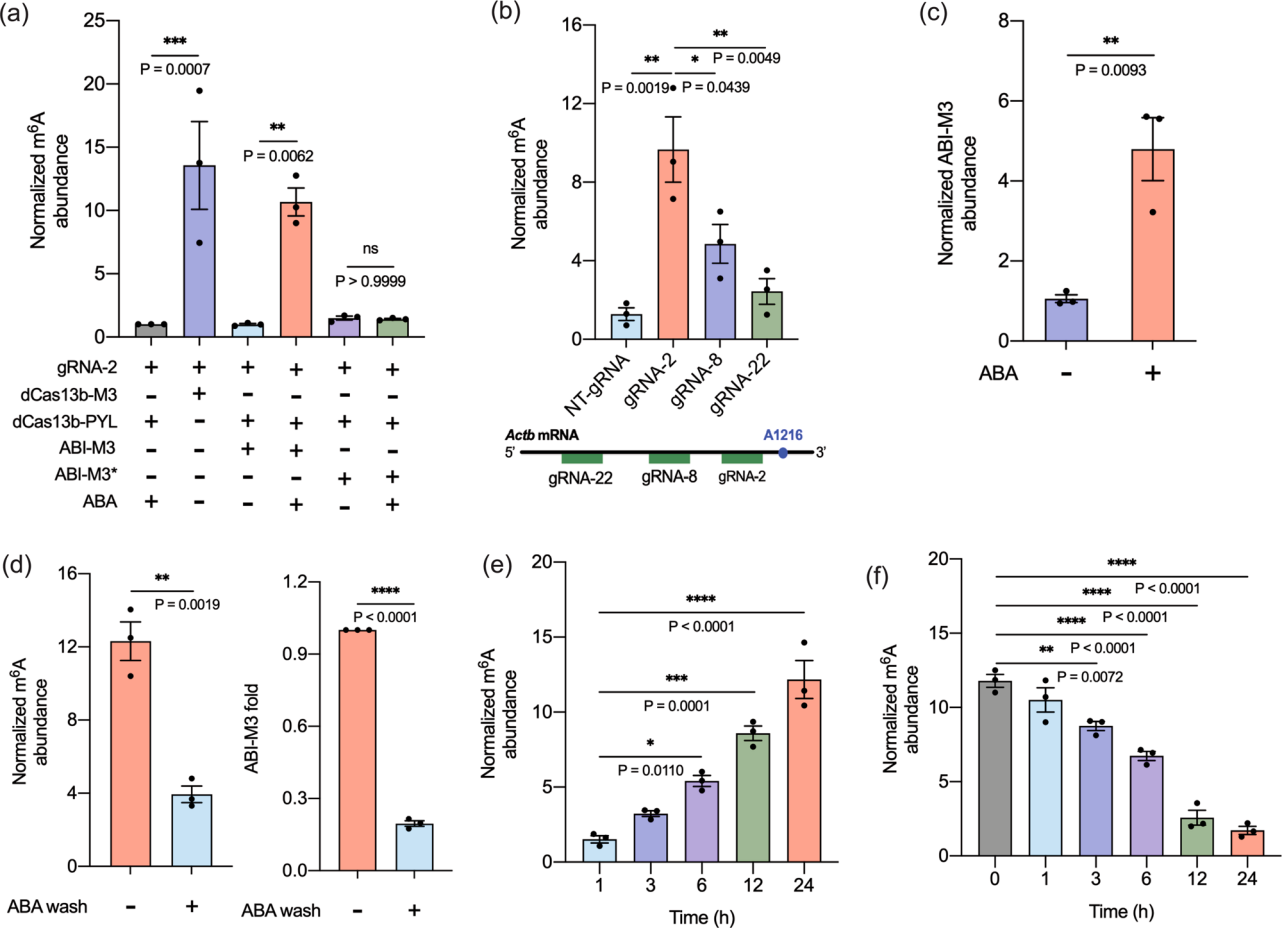
ABA-induced m^6^A writing at the *Actb* mRNA A1216 site in HEK293T cells. **a** The m^6^A level changes under different treatment conditions using the ABA-inducible or non-inducible m^6^A writing system. **b** The m^6^A level changes using the ABA-inducible m^6^A writing system with different gRNAs. **c** The ABI-M3 level changes with or without ABA at the target site. **d** The m^6^A level (left) and the ABI-M3 level (right) change before or after ABA removal (for 24 h). **e** The m^6^A enrichment at different time points after adding ABA. **f** The m^6^A enrichment at different time points after washing out ABA. ABA was added to induce m^6^A writing for 24 h before the time-course experiments of ABA removal. All results were calculated by normalization of data from each sample to that from the condition of dCas13b-PYL plus gRNA-2 and ABA. Values and error bars reflect the mean, s.e.m. of three independent biological replicates. *P* values are shown in the charts are determined by one-way ANOVA. Source data are provided as a Source Data file.

We confirmed the expression and nuclear localization of dCas13b-PYL and ABI-M3 fusion proteins in HEK293T cells by western blotting and immunostaining (Supplementary Figs. 1 and 2). To examine if the designed ABA-inducible editing system can induce m^6^A writing on a specific RNA transcript, we tested the installation of m^6^A at the A1216 site within the 3′- untranslated region (UTR) of *Actb* mRNA. This locus has been reported to have low m^6^A abundance^40^ and has shown to be a validated m^6^A locus in previous m^6^A editing studies^25^. A gRNA (gRNA-2, with its 3′-end two nucleotides (nts) away from the A1216 site on *Actb* mRNA) was used to target dCas13b-PYL. We transfected HEK293T cells with plasmids expressing dCas13-M3, or dCas13-PYL plus ABI-M3 (or ABI-M3*), and gRNA-2 for 24 h and then treated cells with or without ABA (100 μM) for another 24 h. Total RNAs were then isolated from cell lysates and fragmented, followed by the enrichment of m^6^A-containing RNA sequences through immunoprecipitation (IP) using the anti-m^6^A antibody. The levels of m^6^A at the *Actb* A1216 site under different experimental conditions were quantified by reverse transcription and quantitative PCR (RT-qPCR). We observed that the m^6^A level at the *Actb* A1216 region probed by the qPCR primers increased only when ABA was added and when the catalytically active METTL3 was used (Fig. 2a), and the increased m^6^A level was comparable to the constitutively active dCas13-M3 fusion. No m^6^A enrichment was observed when the mutant METTL3 domain was used with or without ABA. To confirm the m^6^A enrichment indeed occurred at the A1216 locus, we performed experiments using the SELECT method to probe the m^6^A level changes specifically at the A1216 site under each editing condition with single-base resolution^41^. We designed 2 synthetic DNA oligonucleotides with PCR adapters that were complementarily annealed to the *Actb* RNA surrounding the A1216 region but left a single nucleotide gap opposite to the A1216 locus. The presence of m^6^A at A1216 prohibited the single-base elongation of DNA polymerases and the nick ligation activity of ligases, resulting in reduced full-length ligation products (quantified by qPCR) when compared to the unmethylated RNA^41^. From the SELECT experiments specifically design to probe the A1216 locus, we indeed observed increased m^6^A levels at A1216 only when ABA was added to the inducible m^6^A writing system or when dCas13-M3 was used (Supplementary Fig. 3). In addition, we investigated the dosage response of ABA in inducing m^6^A writing. We observed that the m^6^A levels increased with increasing ABA levels (Supplementary Fig. 4). Minimal changes were observed when using ABA concentrations higher than 100 μM. These results indicated that 100 μM ABA was an optimal concentration for inducing m^6^A editing, and therefore, was used in the following studies.

To examine if the gRNA targeting locus affects the editing efficiency and to confirm the editing was achieved by transcript/site-specific targeting, we cloned additional gRNAs designed to target different sites with varying distances from the A1216 site, including gRNA-8 and gRNA-22 (with their 3′-end 8 or 22 nucleotides (nts) away from A1216), as well as a non-targeting gRNA (NT-gRNA) as a negative control^25^. After transfecting cells with plasmids expressing dCas13-PYL, ABI-M3, and individual gRNAs for 24 h, ABA was added for another 24 h. RNAs were purified and analyzed for m^6^A level changes as described above. We observed that all tested gRNAs, except NT-gRNA, resulted in m^6^A enrichment at the *Actb* A1216 site (Fig. 2b), although with different levels of increase. Based on the increased m^6^A fold changes, gRNA-2, which targeted a region closest to the A1216 site, was chosen and used in the following studies.

To confirm that ABI-M3 was recruited upon ABA addition to the targeted region on *Actb* mRNA to enable m^6^A writing, we transfected cells with dCas13-PYL, ABI-M3, and gRNA-2 expressing plasmids and treated cells with or without ABA, followed by performing cross-linking immunoprecipitation (CLIP) experiments with cell lysates (using anti-FLAG antibody) and RT-qPCR assays to quantify the ABA-dependent enrichment of ABI-M3. We observed that the level of ABI-M3 was increased at the targeted site on *Actb* mRNAs only when ABA was added (Fig. 2c), which verified the ABA-dependent recruitment of the METTL3 domain.

### Reversibility and the temporal control of m^6^A writing by the ABA-inducible system

To investigate the reversibility of the induced m^6^A writing and ABI-M3 recruitment, HEK293T cells were transfected with dCas13b-PYL, ABI-M3, and *Actb* gRNA-2 for 24 h and treated with ABA for another 24 h. Cells were then washed with fresh cell culture media (without ABA) to remove ABA and incubated for 24 h before cells were lysed and processed as described above to quantify the level changes of m^6^A and ABI-M3 at the *Actb* A1216 site. We observed that both m^6^A and ABI-M3 levels were significantly reduced after ABA removal (Fig. 2d), confirming that the induced m^6^A writing and ABI-M3 recruiting were reversible. To obtain kinetic information regarding the inducing and reversing processes of this m^6^A writing system, we performed time-course experiments monitoring changes of the m^6^A levels at the *Actb* A1216 site at different time points after adding ABA (Fig. 2e), or removing ABA for different time periods after a 24-h ABA induction (Fig. 2f). We observed a rapid induction of m^6^A writing at the *Actb* A1216 site within 3 h, and the m^6^A levels continued to increase within the 24-h observation period. After ABA was removed, the m^6^A levels were significantly reduced in 3 h and eventually reached the background level within 24 h. These results demonstrated that the ABA-inducible m^6^A writing system enabled precise temporal control for both inducing and reversing m^6^A modification by the addition or removal of ABA.

### Feasibility of the ABA-inducible m^6^A editing system on other RNA transcripts

**Fig. 3:**
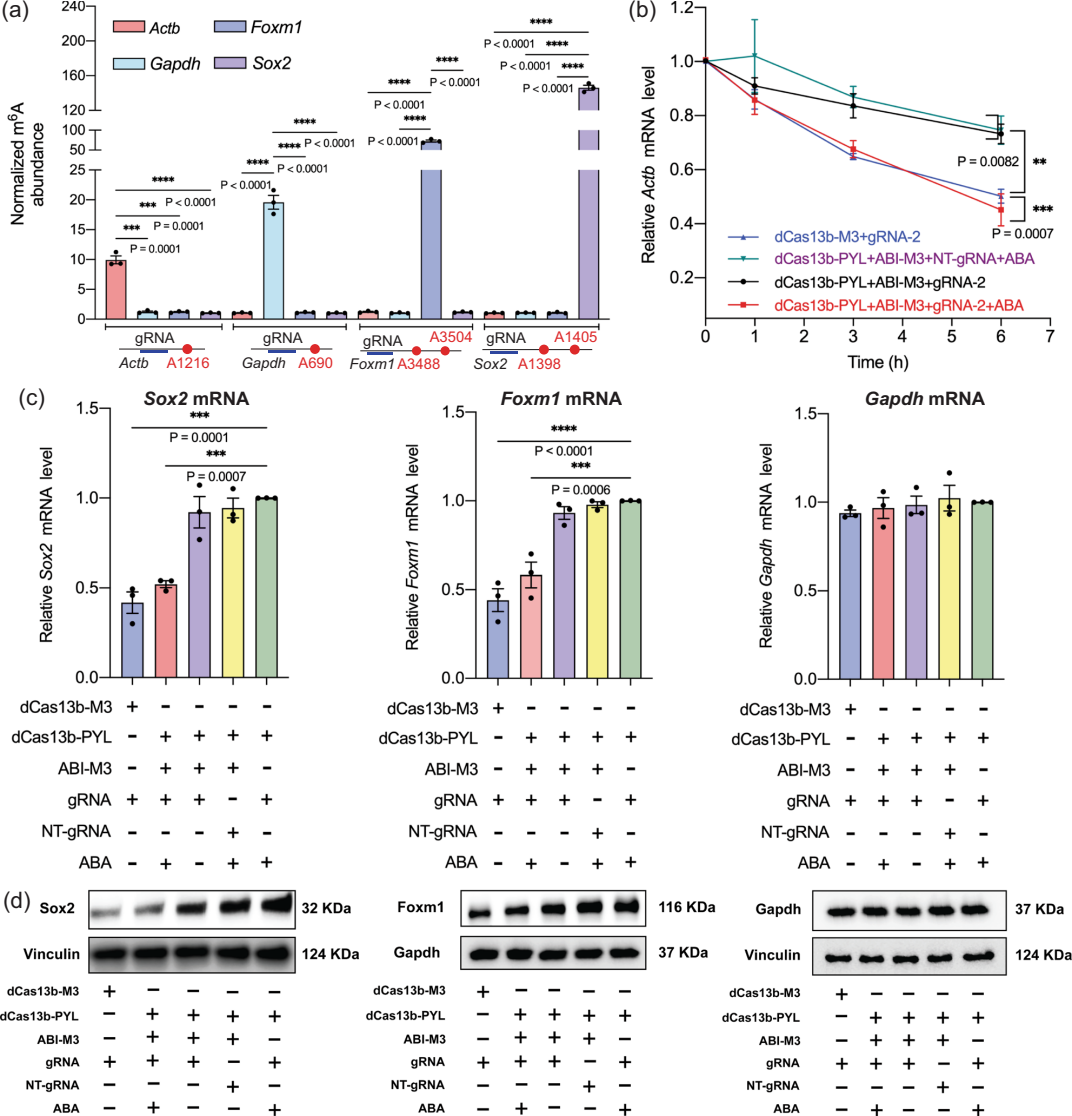
ABA-induced m^6^A writing on different RNAs and the biological effects on RNA stability and translation of installed m^6^A. **a** The relative m^6^A level changes at different mRNA sites when using different mRNA-specific gRNAs for editing. **b** The relative abundance of *Actb* mRNAs in HEK293 cells at different time points after actinomycin D treatment under different m^6^A writing conditions. Gapdh was used as the internal control gene. **c** The relative abundance of *Sox2*, *Foxm1*, and *Gapdh* mRNAs in HEK293 cells under different m^6^A writing conditions. The mRNA levels were quantified after 6-h actinomycin D treatment. *P* values are shown in charts determined by one-sample *t* and Wilcoxon test. **d** Western blotting of *Sox2*, *Foxm1*, and *Gapdh* protein levels (top panel) under different m^6^A writing conditions. Endogenous *Vinculin* or *Gapdh* were used as the internal control (lower panel). All results of groups in **a**–**c** with different treatments were normalized to the group of dCas13b-PYL with gRNA-2 and ABA treatment. Values and error bars reflect the mean, s.e.m. of three independent biological replicates. *P* values shown in the charts are determined by one-way ANOVA. Three independent trials were performed for **d** and representative images were shown. Source data are provided as a Source Data file.

To test if this ABA-inducible m^6^A writing platform can be applied to edit m^6^A on other RNA transcripts, we cloned DNA constructs of gRNAs targeting other RNAs at sites with low m^6^A levels but susceptible to m^6^A writing, including *Gapdh* A690, *Foxm1* A3488, and A3504, *Sox2* A1398 and A1405 sites^25^. We transfected cells with dCas13b-PYL, ABI-M3 combined with corresponding gRNAs of *Actb*, *Gapdh*, *Foxm1,* or *Sox2*, and treated cells with ABA. The samples were processed and analyzed as described above to quantify the m^6^A level at each site under different gRNA targeting conditions. When using the specific gRNA targeting one of the four tested RNA sites, we expected that only the targeted RNA site showed an increased m^6^A level but not at any other tested sites. As expected, when the *Actb* gRNA was used, we observed that only *Actb* A1216 site showed enrichment of m^6^A but not at the sites on *Gapdh*, *Foxm1,* or *Sox2* (Fig. 3a). The same observations were found in the cases of using gRNAs for *Gapdh*, *Foxm1,* and *Sox2*, respectively. These results suggested that this ABA-inducible m^6^A writing system can be robustly applied to editing m^6^A on different RNAs by choosing different gRNAs and the editing was selective among the tested editing studies. We expect that the off-target editing rate of this method will be similar to other dCas13b or dCas9-based methods, which have been reported to have a ~2.8% off-site editing rate out of all RNA transcripts through comprehensive RNA sequencing studies^24,25^.

### Induced *Actb* A1216 m^6^A writing reduced *Actb* mRNA stability

The m^6^A at the *Actb* A1216 site has been shown to reduce its stability^24^. To examine if the artificially installed m^6^A through the inducible editing method was biologically relevant and resulted in the expected downstream effects, we investigated the stability of *Actb* mRNA under different m^6^A editing conditions in HEK293T cells. We transfected HEK293T cells with plasmids expressing dCas13-M3, or dCas13-PYL plus ABI-M3, with gRNA-2 or NT-gRNA for 24 h. After treating cells with or without ABA for another 24 h, actinomycin D, an RNA polymerase inhibitor, was added to block the synthesis of new RNAs^14^. At different time points after actinomycin D treatment, cells were harvested and subjected to RT-qPCR assays to quantify the *Actb* mRNA abundance under different experimental conditions. We observed that the stability of *Actb* mRNA was significantly reduced when the inducible m^6^A writing system was supplied with ABA and when gRNA-2 was used (but not without ABA or with NT-gRNA) (Fig. 3b). The observed reduction in *Actb* mRNA stability caused by the ABA-induced m^6^A writing showed a reduction level similar to the results when using the non-inducible dCas13-M3 system. To confirm that reversing the m^6^A writing can also lead to the reversal of m^6^A-dependent destabilization of *Actb* mRNA, we repeated the experiments described above, except that, after the 24-h ABA induction period, we removed ABA and incubated for another 24 h (or without removal as the control) before adding actinomycin D. We observed that when ABA was removed, the *Actb* mRNA indeed become stabilized and returned to the endogenous level (Supplementary Fig. 5). These results confirmed that the ABA-induced m^6^A writing system-generated functional RNA modification changes that resulted in the expected effect, supporting that the ABA-controlled m^6^A editing system can be a valid tool for studying m^6^A cellular functions.

### Investigation of the impact of m^6^A on other mRNAs

We next applied the inducible m^6^A writing system to examine the effects of m^6^A located in different regions on other mRNAs, including the *Sox2* A1398 and A1405 (3′-UTR), *Foxm1* A3488 and A3504 (3′-UTR), and *Gapdh* A690 (gene body) sites, where the function of m^6^A have not been examined. We specifically investigated the effects of m^6^A on RNA transcript stability and translation, which are known to be affected by the m^6^A modification^1^. We applied the inducible system to install m^6^A at the indicated sites on these mRNAs and determined the relative mRNA levels as an indication for transcript stability (as described above) or the protein levels of each mRNA product for the effects on translation (by western blotting) under different m^6^A writing conditions. We observed that the writing of m^6^A at these 3′-UTR sites on *Sox2* and *Foxm1* caused destabilization of transcripts and decreased RNA levels (Fig. 3c), similar to the effects of m^6^A within 3′-UTR observed on other mRNAs^24^. We also observed reduced *Sox2* and *Foxm1* protein levels with m^6^A installed (Fig. 3d), which we believed were due to reduced mRNA levels of these transcripts. On the contrary, the methylation on *Gapdh* A690 (located in the gene body) did not result in any changes in RNA levels (Fig. 3c), which was consistent with previous observations^25^. It has been shown that m^6^A in the gene body can have different effects on different transcripts, including promoting or impeding translation^15,42,43^. However, we did not observe any effects on *Gapdh* translation with m^6^A installed at *Gapdh* A690 (Fig. 3d). More future experiments will be required to determine the biological function of m^6^A at the *Gapdh* A690 site. Nevertheless, the context-dependent variations of m^6^A function highlight the need for precision RNA modification editing tools as in this study.

### The ABA-inducible m^6^A erasing system

**Fig. 4:**
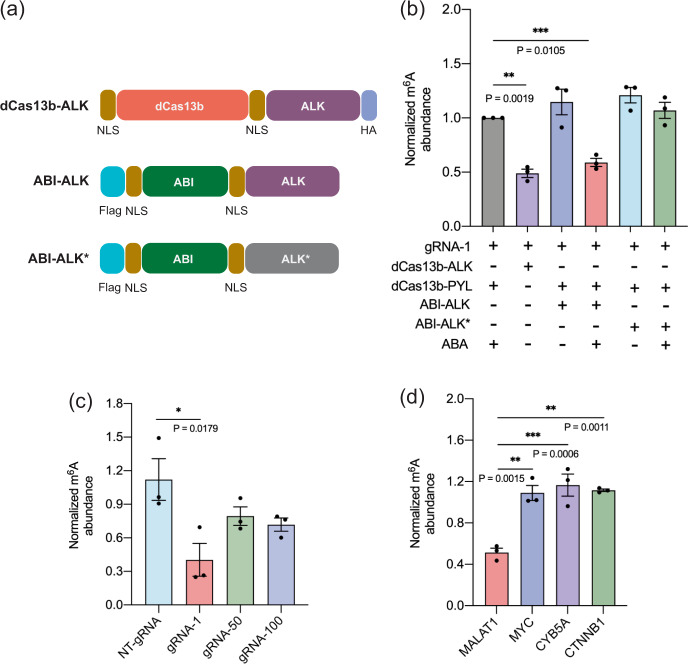
ABA-induced m^6^A erasing at the *MALAT1* RNA A2577 site in HeLa cells. **a** DNA constructs for the ABA-inducible m^6^A erasing system, including dCas13b-ALKBH5, ABI-ALK (with the wild-type ALKBH5), and ABI-ALK* (ALKBH5 with H204A and H267A mutations). **b** The m^6^A level changes under different editing conditions using the ABA-inducible or non-inducible m^6^A erasing system. **c** The m^6^A level changes using the ABA-inducible m^6^A erasing system with different gRNAs. **d** The m^6^A level changes at different RNA sites (*MALAT1 A2577, MYC* A5553, *CYB5A* A135, *CTNNB1* A126) using an inducible m^6^A erasing system targeting the *MALAT1* A2577 site. All results of groups in **a**–**c** with different treatments were normalized to the group of dCas13b-PYL with gRNA-1 and ABA treatment. Values and error bars reflect the mean, s.e.m. of three independent biological replicates. *P* values are shown in the charts are determined by one-way ANOVA. Source data are provided as a Source Data file.

After validating the inducible m^6^A writing system, we investigated the feasibility to apply the same strategy for inducible m^6^A erasing. We expected that by recruiting an m^6^A eraser instead of a writer, ABA-controlled m^6^A erasing can be achieved. The A2577 site on the nuclear non-coding RNA *MALAT1*, known to have a high m^6^A abundance in HeLa cells^40^, was used to test the ABA-inducible m^6^A erasing system. We cloned fusion proteins of ABI-ALKBH5 containing either the catalytic core domain of ALKBH5 (ABI-ALK)^44^ or the inactive H204A/H267A mutant^45^ (ABI-ALK*) (Fig. 4a). The constitutively active dCas13b-ALKBH5 fusion (dCas13b-ALK) and a gRNA (gRNA-1) designed to target the *MALAT1* A2577 site-based on previous studies^27^, were also cloned. We confirmed the expression and subcellular localization of dCas13b-PYL and ABI-ALKBH5 in Hela cells (Supplementary Figs. 6 and 7). To test the ABA-induced m^6^A erasing at the *MALAT1* A2577 site, we transfected Hela cells with plasmids encoding the dCas13-ALK, or dCas13-PYL plus ABI-ALK (or ABI-ALK*) and *MALAT1* gRNA-1 for 24 h and then treated cells with or without ABA (100 μM) for another 24 h. The resulting cell samples were processed and analyzed by m^6^A IP and RT-qPCR assays as described above to quantify the m^6^A levels under different conditions. We observed that m^6^A demethylation at the *MALAT1* A2577 site occurred only when ABA was added to the inducible system and only when the catalytically active ALKBH5 domain was used (Fig. 4b). No m^6^A erasing was observed without ABA or when mutant ALKBH5 was used. The level of induced m^6^A reduction was comparable to the erasing caused by the non-inducible dCas13b-ALK, indicating that the ABA-induced recruiting of ALKBH5 activity and the m^6^A erasing were effective. To confirm the editing occurred at the A2577 locus, we performed SELECT experiments to probe the m^6^A specifically at the *MALAT1* A2577 site and observed decreased m^6^A levels at this locus only when ABA was added to the inducible m^6^A erasing system or when dCas13-ALK was used (Supplementary Fig. 8). These results confirmed that the ABA-inducible m^6^A editing strategy can be extended to control m^6^A erasing by recruiting proper enzymatic activity.

To examine the effects of locations that gRNAs targeted in the inducible m^6^A erasing system, we cloned additional gRNAs targeting 50 or 100 nts away from the A2577 site (gRNA-50 and gRNA-100) and compared the editing efficiency using different gRNAs in experiments similar to those described above. We observed that although all these gRNAs led to m^6^A level decreases, using *MALAT1* gRNA-1 resulted in more effective m^6^A erasing than other gRNAs (Fig. 4c). On the contrary, the m^6^A level did not change when using NT-gRNA. These results showed that gRNA targeting locations or the distance to the m^6^A site could be important for editing efficiency.

To investigate the selectivity of the ABA-induced m^6^A erasing system, we transfected each component of ABA-inducible m^6^A erasing system with *MALAT1* gRNA-1 in Hela cells. After adding ABA for 24 h, cells were harvested and total RNA was extracted, followed by m^6^A IP and RT-qPCR assays. We observed that only m^6^A A2577 site showed a significant decrease in m^6^A level, but not at other reported sites with high m^6^A levels including *MYC* A5553, *CYB5A* A135, and *CTNNB1* A126 (Fig. 4d)^27,28^. This result suggested that the ABA-inducible m^6^A erasing system can robustly and selectively remove m^6^A.

### Light-inducible m^6^A writing by incorporating the photo-caged ABA

**Fig. 5:**
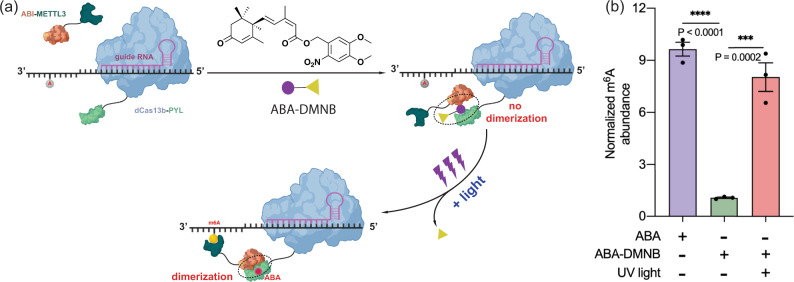
Light-induced m^6^A writing at the *Actb* mRNA A1216 site in HEK293T cells. **a** The light-inducible m^6^A writing system incorporated photo-caged ABA-DMNB that can only be activated by UV light to regenerate functional ABA, which recruits ABI-M3 to enable m^6^A writing. **b** The relative m^6^A levels at the *Actb* mRNA A1216 site were edited by the inducible m^6^A writing system using ABA or ABA-DMNB with or without light irradiation. All results of groups with different treatments were normalized to the group of dCas13b-PYL with gRNA-2 and ABA treatment. Values and error bars reflect the mean, s.e.m. of three independent biological replicates. *P* values shown in the charts are determined by one-way ANOVA. Source data are provided as a Source Data file.

A light-activatable m^6^A editing system has been reported using light-inducible heterodimerization protein pairs^26^, which demonstrated that light can be utilized to control m^6^A editing. To introduce the light control capacity in the ABA-CIP-based m^6^A editing platform, we incorporated the photo-caged ABA that allowed the activation of ABA, and therefore, inducing the RNA m^6^A editing, by light. We have shown that ABA can be caged with sensor units responsive to different stimuli and ABA can only be activated to induce CIP-controlled effects when exposed to the corresponding signals including light^46–48^. To create a light-inducible m^6^A editing system, we used 4,5-dimethoxy-2-nitrobenzyl (DMNB)-caged ABA, which can be uncaged by UV light^48^. It has been reported that caged ABA-DMNB cannot induce dimerization between PYL and ABI^48^, therefore, no m^6^A editing was expected with ABA-DMNB treatment. However, with UV light irradiation, the DMNB group can be removed to generate functional ABA and subsequently achieve m^6^A editing (Fig. 5a).

We synthesized ABA-DMNB as previously reported^48^ and its UV light-dependent uncaging to generate ABA was confirmed using HPLC analysis (Supplementary Fig. 9). To test light-inducible m^6^A editing, we tested using ABA-DMNB and light to control m^6^A writing at the *Actb* A1216 site. We transfected cells with constructs expressing dCas13b-PYL, ABI-M3, and *Actb* gRNA-2 for 24 h, and treated cells with ABA or ABA-DMNB and with or without UV light irradiation (2 min) for another 24 h before cells were harvested to quantify the m^6^A level. We observed that the m^6^A level increased only when cells were treated with ABA or with ABA-DMNB plus UV light, but not with ABA-DMNB without light (Fig. 5b). These results confirmed that by incorporating a photo-caged CIP inducer, ABA-DMNB, a light-controlled m^6^A editing platform can be created, which provides further spatiotemporal controls than using the chemical ligand alone, and can potentially provide dosage-dependent control of effects^48^, which is difficult to achieve using light-inducible dimerization proteins.

## Discussion

The RNA modification has emerged as another layer of epigenetic regulatory mechanism and plays a key role in fundamental biological processes and human disease development. The field has benefited from the rapid development of CRISPR-dCas-based editing methods. To further advance the development of these critical tools in this important research direction, we established an ABA-inducible RNA modification editing platform and showed that the writing and erasing of RNA m^6^A modification can be controlled by cell-permeable CIP inducer ABA. We showed that these induced m^6^A editing events were temporal and RNA transcript-specific with efficiencies similar to the non-inducible editing systems. We further demonstrated that by incorporating the photo-caged ABA, a light-inducible m^6^A editing system can be developed. Using this tool, we examined the function of m^6^A on different regions of selected mRNAs and observed differential effects. We expect that this readily customizable inducible platform can be applied to edit other RNA modifications by replacing METTL3 or ALKBH5 domains used in this study with enzymes responsible for other RNA modifications (e.g., m^5^C, m^1^A, and others)^2^. This strategy and platform will expand the existing toolbox for studying the dynamic biological function of RNA modifications in biological or disease processes. By caging ABA with other sensor units other than photo-caging groups^46,47^, these RNA epigenetic editing events can be coupled to various cellular signals, which can lead to new synthetic biology strategies in engineering mammalian cells for research or therapeutic purposes.

## Methods

### Plasmid design and molecular cloning

Chemically inducible m^6^A editors were constructed by replacing dCas13b or METTL3 (M3) from pCMV-dCas13-M3nls (Addgene, #155366) with ABI or PYL. Typically, PYL or ABI primers ordered from Thermo Fisher Scientific were used to amplify PYL or ABI fragments with Q5 High-Fidelity 2x Master Mix (New England BioLabs, #M0492S), using PCR instrument (C1000 Touch Thermal Cycler). The PCR fragments were then inserted into linearized pCMV-dCas13b-M3nls through T4 DNA ligation (New England BioLabs, #M0202S,). The guide RNAs were cloned via the golden-gate cloning method by inserting the target sequence into the guide RNA backbone, pC0043-PspCas13b-crRNA (Addgene, #103854). All the plasmids were verified with Sanger sequence (GENEWIZ lnc.).

### Mammalian cell lines

HEK293T cells and HeLa cells were gifts from Dr. Gerald R Crabtree at Stanford University Medical School, and were cultured in Dulbecco’s Modification of Eagle’s Medium (Thermo Fisher Scientific, #11965118) with the addition of 10% fetal bovine serum (Thermo Fisher Scientific, #A4766801) and 1% penicillin/streptomycin (Sigma-Aldrich, #P4333). Both HEK293T cells and HeLa cells were incubated at 37 °C with 5% CO_2_. Different cell passages were used for each biological replicate.

### Western blotting

HEK293T cells or Hela cells were seeded in six-well plate and transfected with 1.5 µg of each plasmid at 75% confluency. After 24 h, cells were washed with ice-cold phosphate-buffered saline (PBS) twice and lysed with 200 µL of ice-cold RIPA buffer (Thermo Fisher Scientific, #89901) supplemented with 2 µL protease inhibitor cocktail (Thermo Fisher Scientific, #78430). 20 µg of lysate from each sample was then denatured at 95 °C for 5 min and loaded into each well of SDS-PAGE gel. After separating proteins by running gel at a constant current of 120 mA for 100 min, proteins were transferred from gel to the membrane at a constant current of 10 mA at 4 °C overnight. The membrane was first blocked with 5% milk in TBST (TBS + 0.5% Tween-20) at room temperature for 1 h and incubated with HA Tag Monoclonal Antibody (2-2.2.14) (Invitrogen, #26183, 1:1000 dilution) or Flag Tag Monoclonal Antibody (M2) (Sigma, #F1804, 1:1000 dilution) or anti-Gapdh antibody GAPDH (D16H11) XP® Rabbit mAb (Cell Signaling and Technology, #5174, 1:1000 dilution) at 4 °C overnight. After washing with TBST three times, the membrane was incubated with anti-rabbit IgG HRP-linked antibody (Cell Signaling Technology, #7074, 1:2500 dilution) or anti-mouse IgG HRP-linked antibody (Cell Signaling Technology, #7076, 1:2500 dilution) at room temperature for 1 h. Finally, the membrane was washed with TBST three times and then imaged on ChemiDoc^TM^ MP Image System (Bio-Rad Laboratories). *Gapdh* was used as the reference control.

### Immunofluorescence imaging

HEK293T cells or Hela cells were seeded in six-well plate and transfected with plasmids at nearly 70% confluence. At 24 h post-transfection, cells were fixed with 4% paraformaldehyde for 10 min. Subsequently, the above-fixed cells were treated with 0.1% Triton™ X-100 for 15 min for permeabilization and then blocked with 1% bovine serum albumin (BSA) for 1 h at room temperature. After blocking, cells were incubated with HA Tag Monoclonal Antibody (2-2.2.14) (Invitrogen, #26183) or Flag Tag Monoclonal Antibody (M2) (Sigma, F1804, 1:1000 dilution)at 4 °C overnight and then with Alexa Fluor Plus 488 (Thermo Fisher Scientific, #32723) for 1 h at room temperature. In addition, 4′,6-diamidino-2-phenylindole was added to fixed cells and incubated for 30 min at room temperature followed by washing three times with PBS. Fluorescence images were obtained using the Fluorescence microscope.

### RNA immunoprecipitation (RIP) for m^6^A enrichment

HEK293T cells or Hela cells were first seeded on 10-cm dish in a culture medium. At ~75% confluency after plating, cells were transfected with 6 µg of dCas13b-PYL plasmid and 6 µg of ABI-X (X equals to M3, M3*, ALK, or ALK*) plasmid and 4 µg of PspCas13b guide RNA plasmid using Lipofectamine 2000 (Thermo Fisher Scientific, #11668019) according to manufacturer’s protocol. Spacer sequences of PspCas13b guide RNA were listed in Supplementary Table 1. For control groups, only 6 µg of dCas13b-PYL, dCas13b-M3, or dCas13b-ALK plasmid were transfected with 4 µg of the related PspCas13b guide RNAs. Unless otherwise noted, after 24 h post-transfection, cells were treated with different concentrations of ABA in each inducible system group and incubated for another 24 h before harvest.

After 48 h post-transfection, cells were washed with PBS twice. Total RNA was isolated by TRIzol^TM^ reagent (Thermo Fisher Scientific, #15596026) and purified according to the manufacturer’s protocol. Subsequently, the total RNA was fragmented in freshly prepared RNA fragmentation buffer (10 mM Tris-HCl pH = 7.0, 10 mM ZnCl_2_) at 94 °C for 1 min. The fragmented RNA was collected. One-tenth volumes of 3 M sodium acetate and 2.5 volumes of 100% ethanol were added to precipitate RNA. The mixture was then incubated at −80 °C overnight. After that, the mixture was centrifuged at 4 °C at 15,000 × *g* for 25 min, then RNA was washed with 75% ethanol, and air-dried for 5–10 min. The fragmented RNA pellet was then resuspended with nuclease-free water and the concentration was measured by Nanodrop (Thermo Fisher Scientific). A portion of fragmented RNA was saved as input samples for RT-qPCR.

m^6^A immunoprecipitation (m^6^A IP) was carried out according to the protocol reported by Dominissini et al.^49^. Typically, 400 µL of reaction mixture containing 120 µg of fragmented RNA, 4 µL of RNasin Plus RNase Inhibitor (40 U/µL, Promega), 4 µL of RVC (200 mM, Sigma), 80 µL of 5× IP buffer (0.5 mL 1 M Tris-HCl (pH 7.4), 1.5 mL 5 M NaCl, 0.5 mL 10% of Igeal CA-630, and 7.5 mL of Nuclease-free water), and 10 µL m^6^A antibody (Cell Signal and Technology, #56593), was incubated with head-over-tail rotation for 2 h at 4 °C. At the same time, 40 µL of protein G magnetic beads (Thermo Fisher Scientific, #10004D) was washed with 1× IP buffer twice. The beads were then resuspended with 1× IP buffer supplemented with BSA (0.5 mg/mL) and incubated head-over-tail for 2 h at 4 °C. After washing with 1× IP buffer twice, the beads were resuspended in each reaction mixture and incubated for at least 4 h at 4 °C.

Then, beads were washed with 1× IP buffer three times and resuspended in 100 µL of elution buffer (90 µL 5× IP buffer, 150 µL 20 mM m^6^A salt, 7 µL RNasin Plus, and 203 µL nuclease-free water) with continuous shaking for 1 h at 4 °C. The supernatant was retained. Beads were washed with 100 µL of 1× IP buffer and supernatant was retained again. The elution process was repeated once and the supernatant was collected together. One-tenth volumes of 3 M sodium acetate and 2.5 volumes of 100% ethanol were added to the above elution mixture. The mixture was then incubated at −80 °C overnight. After that, the mixture was centrifuged at 4 °C, washed with 75% ethanol, and air-dried for 5–10 min. The RNA fragments pulled down were resuspended with nuclease-free water and saved as IP samples for RT-qPCR.

### The SELECT method to determine the m^6^A level at a specific locus

The detection of m6A at targeted sites was based on SELECT method previously developed by Xiao et al.^41^. In all, 1.5 µg total RNA was first mixed with 40 nM Up primer, 40 nM Down primer, and 5 µM dNTP in 17 µL 1v CutSmart buffer. The mixture was then annealed in the following programs: 90 °C (1 min), 80 °C (1 min), 70 °C (1 min), 60 °C (1 min), 50 °C (1 min), and 40 °C (6 min). After that, 17 µL annealing products were incubated with 3 µL enzyme mixture containing 0.01 U Bst 2.0 DNA polymerases, 0.5 U SplintR liagase, and 10 nmol ATP. Subsequently, the final mixture (totally 20 µL) above was incubated at 40 °C for 20 min, denature at 80 °C for 20 min. qPCR was carried out described above. Primers used in SELECT measurement are listed in Supplementary Table 3.

### CLIP for ABI-M3 enrichment

ABI-M3 recruitment assay was performed based on the previously reported method^50^. Typically, HEK293T cells were transfected with 1.5 µg of dCas13b-PYL, 1.5 µg of ABI-M3, and 1.0 µg of *Actb* gRNA-2 in six-well plate. After 24 h post-transfection, ABA or dimethyl sulfoxide (no ABA) was added and cells were incubated for another 24 h. For ABA washout experiments, cells were washed with PBS three times and incubated with fresh Dulbecco’s Modified Eagle Medium (10% FBS) for another 24 h. Cells were then washed with ice-cold PBS (Sigma) and fixed with 0.2% paraformaldehyde (Sigma) in PBS for 15 min at room temperature. After fixation, 125 mM glycine was added to quench cross-linking and incubated at room temperature for another 10 min. Cells were washed with ice-cold PBS twice and harvested by scraping, followed by centrifugation at 800 × *g* for 5 min. The cell pellets were resuspended and lysed with 200 µL of RIPA Buffer (Cell Signaling) supplemented with RNase inhibitor (Promega) and Protease Inhibitor Cocktail (PIC, Thermo Fisher Scientific). Cells were incubated on ice for 10 min and sonicated for 1 min with a 30 s on/30 s off-cycle on a Bioruptor sonicator (Diagenode), followed by centrifugation at 16,000 × *g* for 10 min at 4 °C. The clear supernatant containing lysate was then used for RNA-protein IP. A portion of lysate was saved as an input sample.

For RNA-protein IP, 100 µL of Dynabeads Protein A were washed with 200 µL of wash buffer (PBS with 0.02% Tween-20 (Sigma)). Then, 5 µg of anti-METTL3 antibody (Invitrogen, #15073-1-AP) was added and incubated on a rotator for 10 min at room temperature. After that, beads were carefully washed with wash buffer twice and resuspended in 200 µL of RIPA buffer supplemented with PIC and RNase inhibitor. In all, 100 µL of above lysate was added and rotated at 4 °C overnight. After incubation, beads were washed with wash buffer twice and proteins were digested by Proteinase K (New England BioLabs) and incubated at 55 °C for 2 h. Urea was then added for purpose of denaturation. RNA was purified with RNA Clean & Concentrator-5 kits (Zymo Research). Purified RNA was reverse transcribed and quantified with qPCR described above.

### mRNA stability assay

HEK293T cells or Hela cells were transfected with different components of the ABA-induced m^6^A modification system, followed by adding ABA for 24 h and then with/without removal of ABA for another 24 h before cells were treated with actinomycin D and harvested at different time points (e.g., 0 h, 1 h, 3 h, and 6 h). After washing with PBS, total RNA was isolated by TRIzol^TM^ reagent (Thermo Fisher Scientific, #15596026) and purified according to the manufacturer’s protocol. After reverse transcription, mRNA levels of target transcripts were analyzed by qPCR. *Gapdh* was used as the internal control for Actin analysis and Actin was used as the internal control for *Gapdh*, *Foxm1,* and *Sox2*.

### m^6^A impact on mRNA translation

HEK293T cells seeded in 24-well plate were transfected with different components of the ABA-induced m^6^A modification system. After 24 h incubation, 100 µM ABA was added to the cells and incubated for another 24 h before harvest. Proteins were extracted as mentioned above and 20 µg of whole proteins were loaded for protein gel electrophoresis. *Gapdh*, *Foxm1*, *Sox2,* and internal control proteins including *Vinculin*, *Gapdh* were probed with the corresponding anti-Gapdh antibody (D16H11) (Cell Signaling Technology, #5174, 1:1000 dilution), anti-Foxm1(A-11) (Santa Cruz, #sc-271746, 1:500 dilution), anti-Sox2 (E-4)((Santa Cruz, #sc-365823, 1:500 dilution), anti-Vinculin (E1E9V, 1:1000) (Cell Signaling Technology, #13901, 1:1000 dilution) and corresponding secondary antibodies as described above.

### RT-qPCR

The input RNA samples saved above and RNA after IP samples were used as templates for reverse transcription to synthesize cDNA according to the manufacturer’s protocol (iScript^TM^ cDNA Synthesis Kit, BIO-RAD). The synthesized cDNAs from input and IP samples were mixed with Power SYBR Green PCR Master Mix (Applied Biosystems) for qPCR in 384-well plate in a 7900HT Fast Real-Time PCR System (Applied Biosystems). Primers for qPCR were listed in Supplementary Table 2. All measurements were replicated in a triplet. C_t_ values were obtained from the SDS software (version 2.4, Applied Biosystem). Folds of m^6^A level or ABI-M3 level were normalized to experimental controls and calculated according to 2^−ΔΔCt^ method^51^.

### Statistics and reproducibility

Information regarding error bars, numbers of replicates or samples, and statistical analyses are described in the corresponding figure legends. Representative results of at least three independent experiments are shown unless otherwise indicated.

### Reporting summary

Further information on research design is available in the Nature Research Reporting Summary linked to this article.

## Supplementary information

Supplementary Information

Reporting Summary

## Data Availability

The authors declare that all data supporting the findings of this study are available within the paper and its Supplementary Information files. Source data are provided in this paper.

## Source data

Source Data
