# Supplementary material for: Inducible and reversible RNA N6-methyladenosine editing
Source: Nat Commun. 2022 Apr 12;13:1958. doi: 10.1038/s41467-022-29665-y (PMC9005610; doi:10.1038/s41467-022-29665-y)
Supplement: Supplementary file 1 — Supplementary Information [file 41467_2022_29665_MOESM1_ESM.pdf]

Chemically Inducible and Reversible RNA N^6-Methyladenosine Editing

Huaxia Shi[1], Ying Xu[1], Na Tian[1], Ming Yang[1], Fu-Sen Liang[1*]

([1]Department of Chemistry, Case Western Reserve University, 2080 Adelbert Road, Cleveland, OH 44106, USA)

These authors contributed equally to this work: Huaxia Shi and Ying Xu

Corresponding author: Fu-Sen Liang, fxl240@case.edu

S1. Expression of fusion proteins in the ABA inducible m^{6}A writing system in HEK293T cells.

S2. Subcellular location of fusion proteins in the inducible m^{6}A writing system in HEK293T cells.

S3. The m^{6}A enrichment at the *Actb* A1216 site under different editing conditions determined by the SELECT method.

S4. The dosage response of ABA-induced m^{6}A writing at *Actb* A1216 site.

S5. The reversibility of m^{6}A-dependent *Actb* mRNA destabilization controlled by the ABA-induced m^{6}A writing system.

S6. Expression of fusion proteins in the ABA inducible m^{6}A erasing system in Hela cells.

S7. Subcellular location of fusion proteins in the inducible m^{6}A erasing system in Hela cells.

S8. The m^{6}A enrichment at the *MALAT1* A2577 site under different editing conditions determined by the SELECT method.

S9. Photo-cleavage of ABA-DMNB by UV light in PBS.

Table S1. PspCas13b guide RNA spacer sequences.

Table S2. Primers for RT-qPCR.

Table S3: Primers for SELET assay.

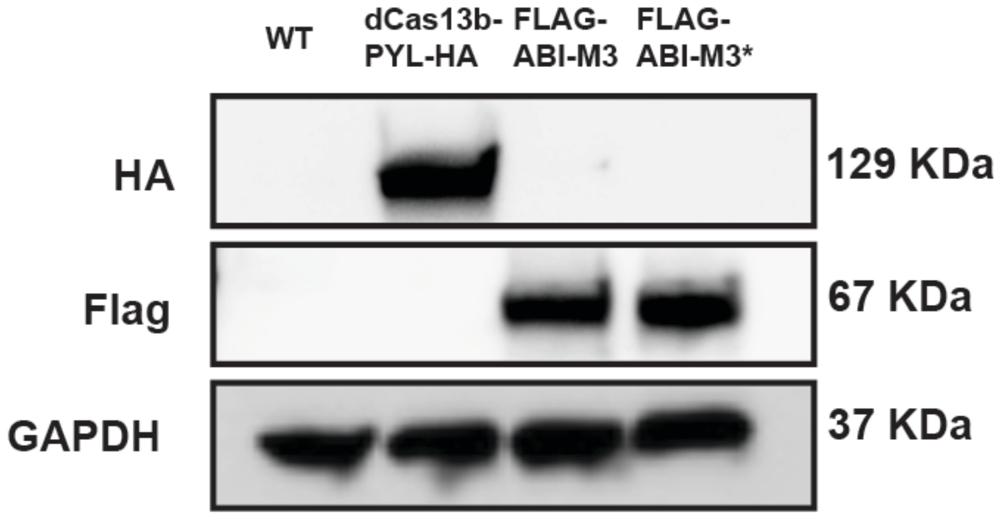

Supplementary Figure 1. Expression of fusion proteins in the ABA inducible m^{6}A writing system in HEK293T cells. Representative western blot of 3xHA-tagged dCas13b-PYL, 3xFLAG-tagged ABI-M3, and 3xFLAG-tagged ABI-M3*. dCas13b-PYL was stained with an anti-HA antibody, ABI-M3 and ABI-M3*were stained with an anti-Flag antibody. *Gapdh* was probed as an internal control. 3 independent trials were performed and representative images were shown. Source data are provided in the source data file.

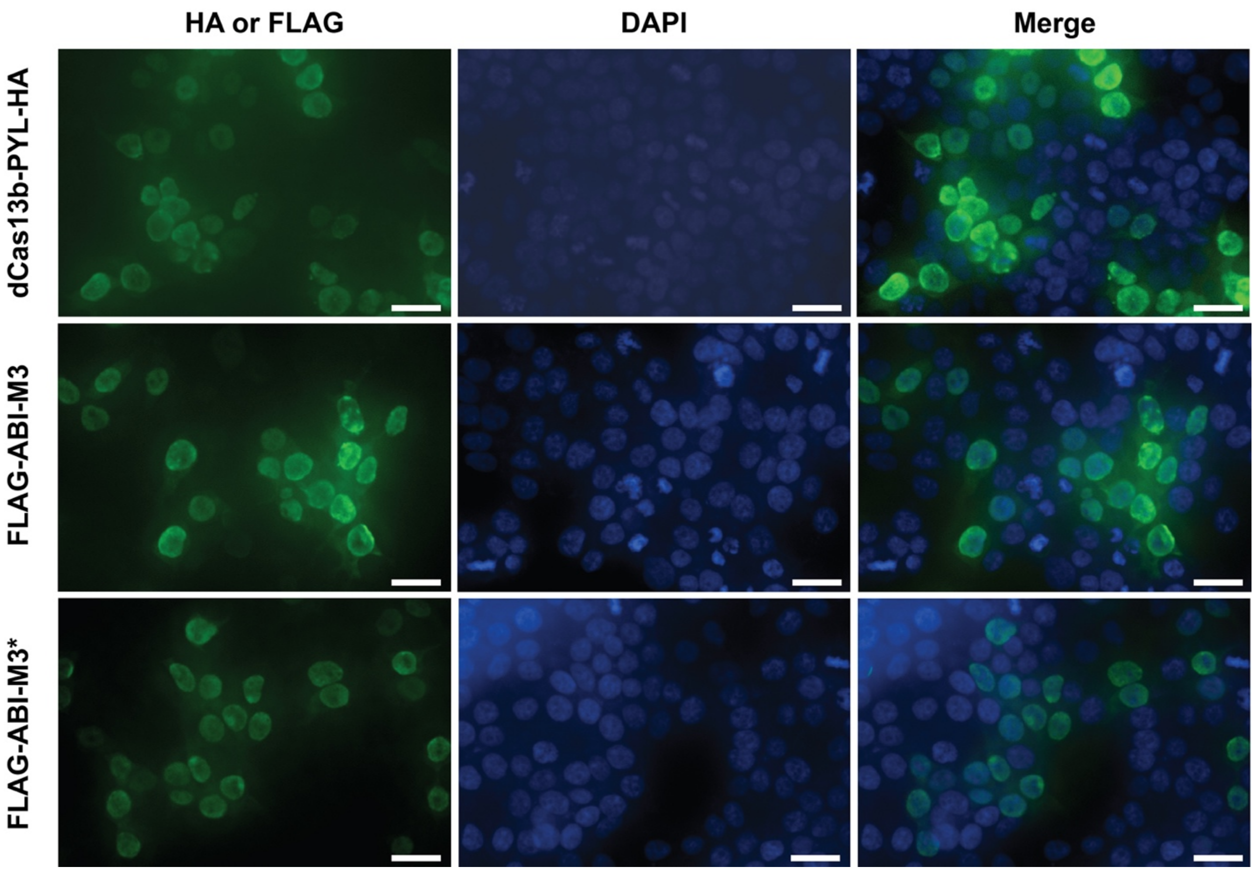

Supplementary Figure 2. Subcellular location of fusion proteins in the inducible m^6A writing system in HEK293T cells. HEK293T cells were transfected with HA-tagged dCas13b-PYL, FLAG-tagged ABI-M3, and FLAG-tagged ABI-M3* and probed by anti-HA or anti-FLAG antibody (green, HA tag or Flag tag; blue: DAPI). Scale bars: 25 μm. These are representative immunofluorescence images from three independent biological repeat experiments.

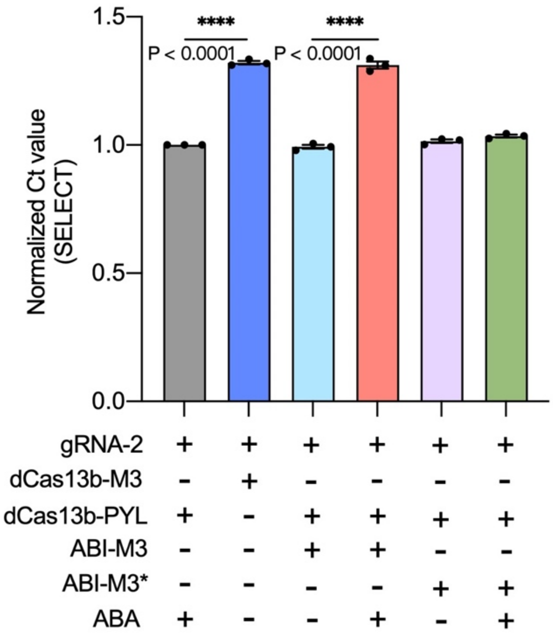

Supplementary Figure 3. The m^{6}A enrichment at the *Actb* A1216 site under different editing conditions determined by the SELECT method. The relative A1216 m^{6}A level under each condition was compared by the relative threshold cycle (Ct) value from qPCR detection. A higher Ct value (i.e., a lower amount of the full-length SELECT product) indicated a higher level of m^{6}A at the probed A1216 site. All results were calculated by normalizing data from each sample to that from the condition of dCas13b-PYL plus gRNA-2 and ABA. Values and error bars reflect the mean, s.e.m. of three independent biological replicates. *P* values shown in charts determined by one-way ANOVA. Source data are provided in the source data file.

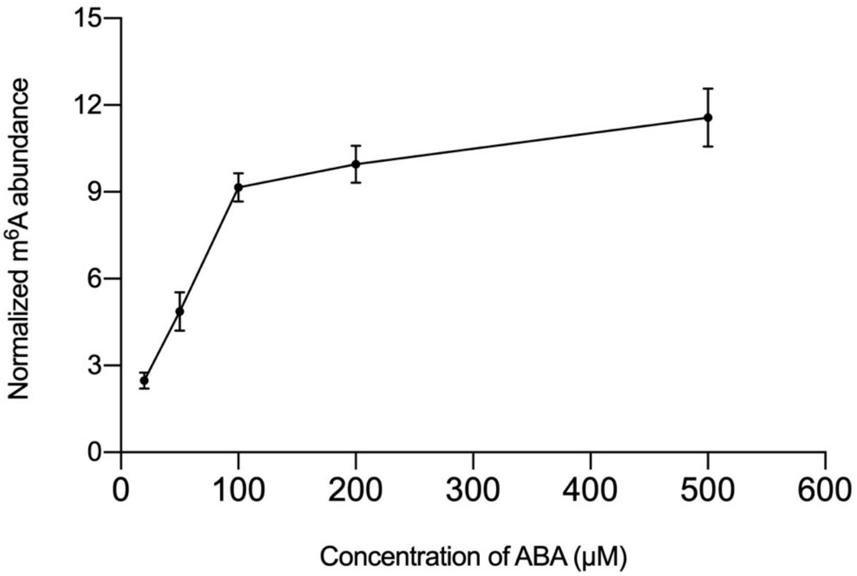

Supplementary Figure 4. The dosage response of ABA-induced m^{6}A writing at *Actb* A1216 site. The m^{6}A enrichment resulted from the inducible m^{6}A writing system under different ABA concentrations. All results were calculated by normalization of data from each sample to that from the condition of dCas13b-PYL plus gRNA-2. Values and error bars reflect the mean, s.e.m. of three independent biological replicates. Source data are provided in the source data file.

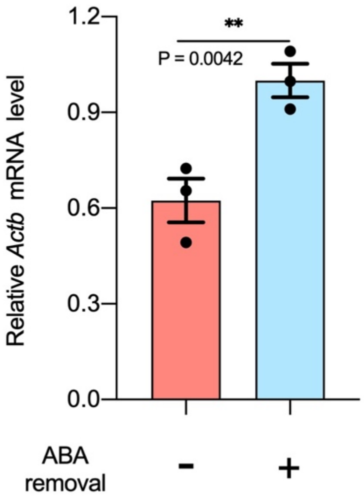

Supplementary Figure 5. The reversibility of m^{6}A-dependent *Actb* mRNA destabilization controlled by the ABA-induced m^{6}A writing system. After the 24-h ABA induction of m^{6}A writing, ABA was removed (+) or not (-) for another 24 h before determining the relative *Actb* mRNA abundance. The mRNA levels were quantified after 6-h actinomycin D treatment. *Gapdh* was used as the internal control. All results were calculated by normalization of data from each sample to that from the condition of dCas13b-PYL plus gRNA-2. Values and error bars reflect the mean, s.e.m. of three independent biological replicates. *P* value shown in charts determined by one-way ANOVA. Source data are provided in the source data file.

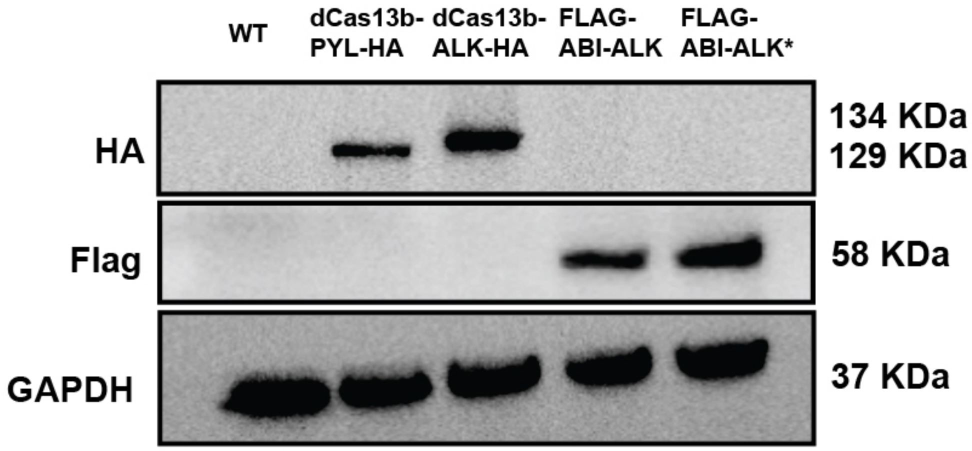

Supplementary Figure 6. Expression of fusion proteins in the ABA inducible m^{6}A erasing system in Hela cells. Representative western blot of 3xHA-tagged dCas13b-PYL, 3xFLAG-tagged ABI-ALK, and 3xFLAG-tagged ABI-ALK*. dCas13b-PYL was stained with an anti-HA antibody, ABI-ALK and ABI-ALK* were stained with an anti-FLAG antibody. *Gapdh* was probed as an internal control. 3 independent trials were performed and representative images were shown. Source data are provided in the source data file.

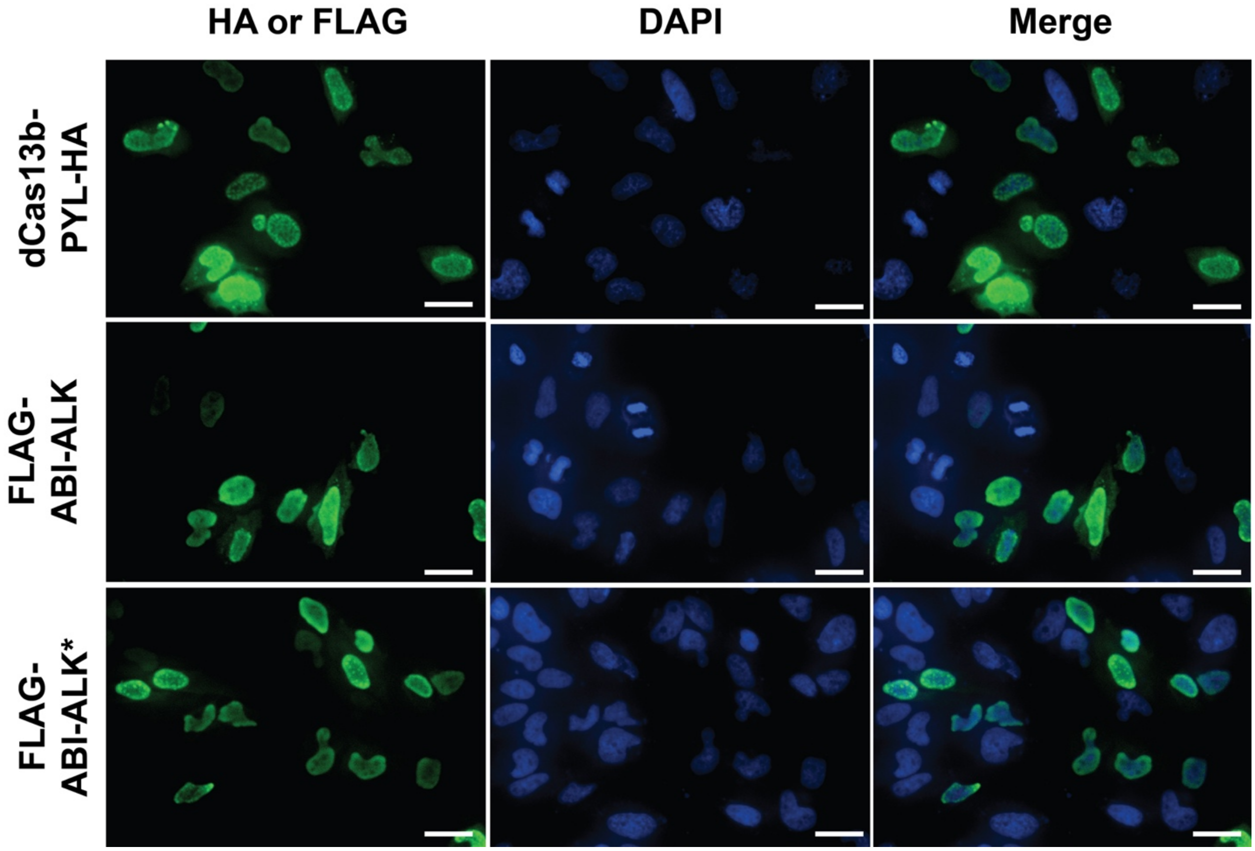

Supplementary Figure 7. Subcellular location of fusion proteins in the inducible m⁶A erasing system in Hela cells. Hela cells were transfected with HA-tagged dCas13b-PYL, FLAG-tagged ABI-ALK, and FLAG-tagged ABI-ALK* and probed by anti-HA or anti-FLAG antibody (green, HA tag or Flag tag; blue: DAPI). Scale bars: 25 μm. These are representative immunofluorescence images from three independent biological repeat experiments.

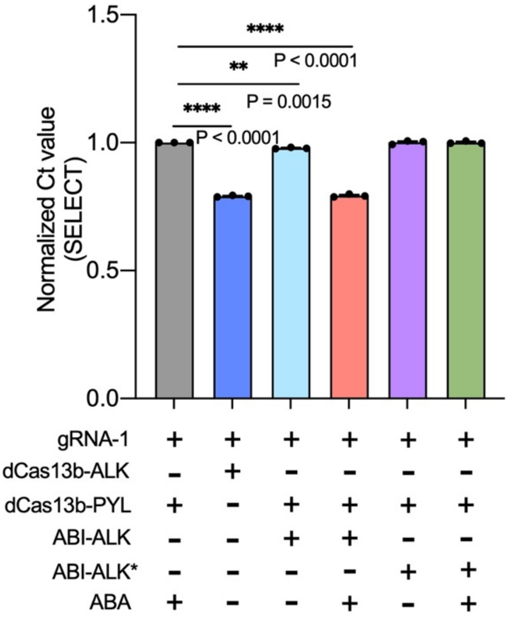

Supplementary Figure 8. The m⁶A enrichment at the *MALAT1* A2577 site under different editing conditions determined by the SELECT method. The relative A2577 m^6A level under each condition was compared by the relative threshold cycle (Ct) value from qPCR detection. A lower Ct value (i.e., a higher amount of the full-length SELECT product) indicated a lower level of m^6A at the probed A2577 site. All results were calculated by normalizing data from each sample to that from the condition of dCas13b-PYL plus gRNA-1 and ABA. Values and error bars reflect the mean, s.e.m. of three independent biological replicates. *P* values shown in charts determined by one-way ANOVA. Source data are provided in the source data file.

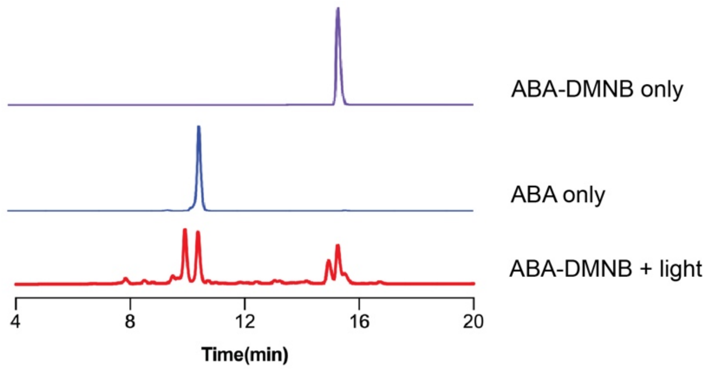

Supplementary Figure 9. Photo-cleavage of ABA-DMNB by UV light in PBS. Peaks of ABA and photo-caged ABA in PBS (25 μM) before and after UV light irradiation monitored by HPLC analysis. Time for UV light irradiation is 1 min. Source data are provided in the source data file.

Supplementary Table 1. PspCas13b guide RNA spacer sequences used in this study. All gRNA spacers were synthesized from Thermo Fisher Scientific.

Actb gRNA-2	TCCATCGTCCACCGCAAATGCTTCTAGGCG
Actb gRNA-8	GGCCCCTCCATCGTCCACCGCAAATGCTTC
Actb gRNA-22	AGTATGACGAGTCCGGCCCCTCCATCGTCC
Gapdh gRNA-8	GGGAAACTGTGGCGTGATGGCCGCGGGGCT
Foxm1 gRNA	ATGTTTCTCTGATAATGTCCCCAATCATAC
Sox2 gRNA	AACGGCACACTGCCCCTCTCACACATGTGA
MALAT gRNA-1	AACGGAAGTAATTCAAGATCAAGAGTAATT
MALAT gRNA-50	GAAGGCCTTAAATATAGTAGCTTAGTTTGA
MALAT gRNA-100	ATTTAAAAAAAACTAAGGCAGAAGGCTTTT

Supplementary Table 2. Primers for RT-qPCR used in this study. All primers were synthesized from Thermo Fisher Scientific.

Actb-m^{6}A-F (5'-3')	AGATGTGGATCAGCAAGC
Actb-m^{6}A-R (5'-3')	TCATCTTGTTTTCTGCGC
Gapdh-m^{6}A-F (5'-3')	CATCACTGCCACCCAGAAGA
Gapdh-m^{6}A-R (5'-3')	CAGTAGAGGCAGGGATGATGTT
Foxm1-m^{6}A-F (5'-3')	TGCCCAGATGTGCGCTATTA
Foxm1-m^{6}A-R (5'-3')	CTTCTCAAGCCTCCACCTGA
Sox2-m^{6}A-F (5'-3')	GGCCATTAACGGCACACTG
Sox2-m^{6}A-R (5'-3')	TCTTTTGCACCCCTCCCATT
MALAT1-m^{6}A-F (5'-3')	CGTAACGGAAGTAATTCAAG
MALAT1-m^{6}A-R (5'-3')	GTCAATTAATGCTAGTCCTC
CYB5A-m^{6}A-F (5'-3')	GTTTTAAGGGAACAAGCTGGAG
CYB5A-m^{6}A-R (5'-3')	TCCACCAACTGGAACTAGAATC
CTNNB1-m^{6}A-F (5'-3')	TGGATTGATTCGAAATCTTGCC
CTNNB1-m^{6}A-R (5'-3')	GAACAAGCAACTGAACTAGTCG
MYC-m^{6}A-F (5'-3')	CAGCTGCTTAGACGCTGGATT
MYC-m^{6}A-R (5'-3')	GTAGAAATACGGCTGCACCGA
Actb-F (5'-3')	TCCCAAGTCCACACAGG
Actb-R (5'-3')	CACGAAGGCTCATCATTCAAA
Gapdh-F (5'-3')	GGTGTGAACCATGAGAAGTATGA
Gapdh-R (5'-3')	GAGTCCTTCCACGATACCAAAG
Foxm1-F (5'-3')	CAATGGCAAGGTCTCCTTCT
Foxm1-R (5'-3')	GGTAGCAGTGGCTTCATCTT
Sox2-F (5'-3')	AGACGCTCATGAAGAAGGATAAG
Sox2-R (5'-3')	TCATGTGCGCGTAACTGT
SELECT-F (5'-3')	ATGCAGCGACTCAGCCTCTG
SELECT-R (5'-3')	TAGCCAGTACCGTAGTGCGTG

Supplementary Table 3: Primers for SELET assay used in this study. All primers were synthesized from Thermo Fisher Scientific.

Atcb A1216	up	tagccagtaccgtagtgcgtgGAAAGGGTGTAACGCAACTAAGTCATAG
	down	5phos/CCGCCTAGAAGCATTTGCGGcagaggctgagtcgctgcat
MALAT1 A2577	up	tagccagtaccgtagtgcgtgGGATTTAAAAAATAATCTTAACTCAAAG
	down	5phos/CCAATGCAAAAACATTAAGTcagaggctgagtcgctgcat

Source data of WB images presented in the supplementary information:

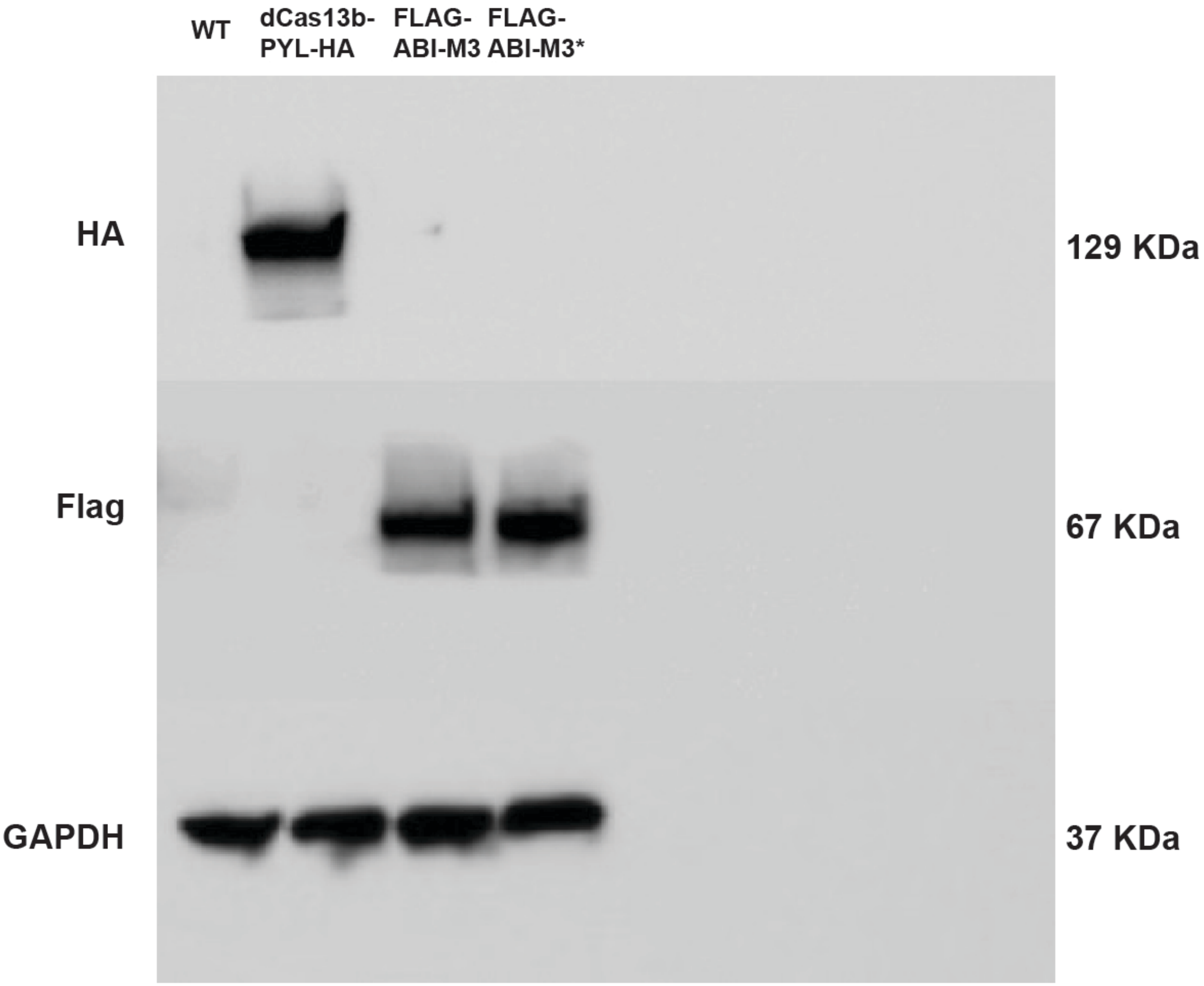

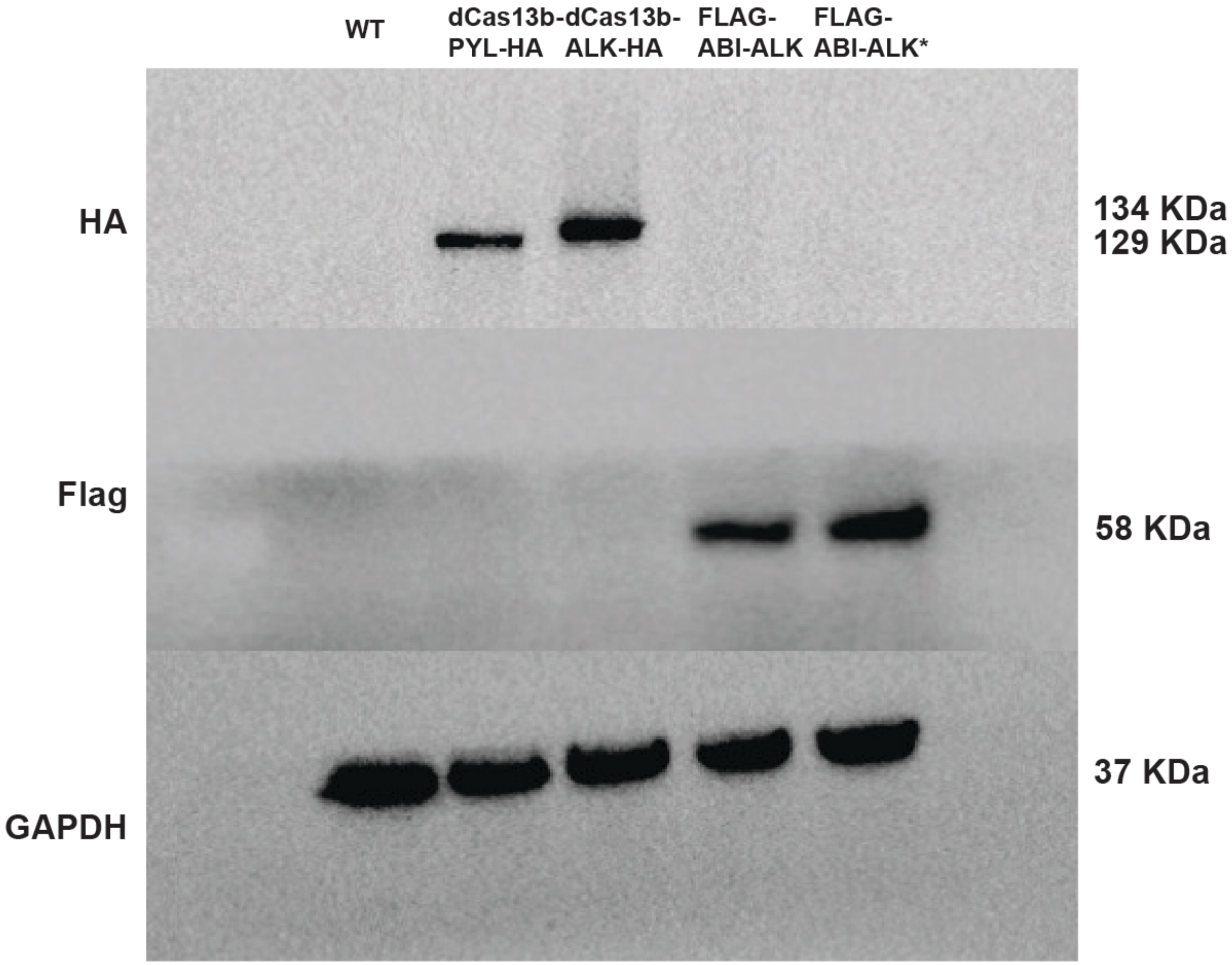
WT
dCas13b-PYL-HA
dCas13b-ALK-HA
FLAG-ABI-ALK
FLAG-ABI-ALK*
HA
Flag
GAPDH
134 KDa
129 KDa
58 KDa
37 KDa
