# Supplementary material for: Inducible and reversible RNA N6-methyladenosine editing
Source: Nat Commun. 2022 Apr 12;13:1958. doi: 10.1038/s41467-022-29665-y (PMC9005610; doi:10.1038/s41467-022-29665-y)
Supplement: Supplementary file 2 — Reporting Summary [file 41467_2022_29665_MOESM2_ESM.pdf]

nature portfolio

Corresponding author(s): Fu-Sen Liang

Last updated by author(s): Mar 2, 2022

Reporting Summary

Nature Portfolio wishes to improve the reproducibility of the work that we publish. This form provides structure for consistency and transparency in reporting. For further information on Nature Portfolio policies, see our Editorial Policies and the Editorial Policy Checklist.

Statistics

For all statistical analyses, confirm that the following items are present in the figure legend, table legend, main text, or Methods section.

n/a	Confirmed	
☐	☒	The exact sample size (n) for each experimental group/condition, given as a discrete number and unit of measurement
☐	☒	A statement on whether measurements were taken from distinct samples or whether the same sample was measured repeatedly
☐	☒	The statistical test(s) used AND whether they are one- or two-sided<br>*Only common tests should be described solely by name; describe more complex techniques in the Methods section.*
☒	☐	A description of all covariates tested
☒	☐	A description of any assumptions or corrections, such as tests of normality and adjustment for multiple comparisons
☐	☒	A full description of the statistical parameters including central tendency (e.g. means) or other basic estimates (e.g. regression coefficient) AND variation (e.g. standard deviation) or associated estimates of uncertainty (e.g. confidence intervals)
☐	☒	For null hypothesis testing, the test statistic (e.g. F, t, r) with confidence intervals, effect sizes, degrees of freedom and P value noted<br>*Give P values as exact values whenever suitable.*
☒	☐	For Bayesian analysis, information on the choice of priors and Markov chain Monte Carlo settings
☒	☐	For hierarchical and complex designs, identification of the appropriate level for tests and full reporting of outcomes
☒	☐	Estimates of effect sizes (e.g. Cohen's d, Pearson's r), indicating how they were calculated

Our web collection on statistics for biologists contains articles on many of the points above.

Software and code

Policy information about availability of computer code

Data collection	qPCR assays were carried on a 7900HT Fast Real-Time PCR System (Applied Biosystems). Western Blotting was imaged on a ChemiDocTM MP Image System (Bio-Rad Laboratories). Immunofluorescence imaging was performed using a BioTek Lionheart FX Automated Microscope (BioTek). SDS software for collecting Ct value (version 2.4, Applied Biosystem).
Data analysis	Graphpad Prism 8.0, Adobe Illustrator 2020

For manuscripts utilizing custom algorithms or software that are central to the research but not yet described in published literature, software must be made available to editors and reviewers. We strongly encourage code deposition in a community repository (e.g. GitHub). See the Nature Portfolio guidelines for submitting code & software for further information.

Data

Policy information about availability of data

All manuscripts must include a data availability statement. This statement should provide the following information, where applicable:

- Accession codes, unique identifiers, or web links for publicly available datasets
- A description of any restrictions on data availability
- For clinical datasets or third party data, please ensure that the statement adheres to our policy

The raw data of all the Figures and Supplementary information are provided in the source data file and are available from the corresponding author upon reasonable request.

March 2021

Field-specific reporting

Please select the one below that is the best fit for your research. If you are not sure, read the appropriate sections before making your selection.

- [x] Life sciences
- [ ] Behavioural & social sciences
- [ ] Ecological, evolutionary & environmental sciences

For a reference copy of the document with all sections, see nature.com/documents/nr-reporting-summary-flat.pdf

Life sciences study design

All studies must disclose on these points even when the disclosure is negative.

Sample size	Sample sizes are indicated in the figure legends, which is determined by similar work published previously.
Data exclusions	No data was excluded.
Replication	Experimental findings were reliably reproduced.
Randomization	Randomization was not performed in our studies. Only cell experiments, but not animal nor clinic trial experiments were performed. All samples for each group were collected under the same condition.
Blinding	Blinding was not used in our studies. Only cell experiments, but not animal nor clinic trial experiments were performed. All samples for each group were collected under the same condition.

Reporting for specific materials, systems and methods

We require information from authors about some types of materials, experimental systems and methods used in many studies. Here, indicate whether each material, system or method listed is relevant to your study. If you are not sure if a list item applies to your research, read the appropriate section before selecting a response.

Materials & experimental systems

n/a	Involved in the study	
☐	☒	Antibodies
☐	☒	Eukaryotic cell lines
☒	☐	Palaeontology and archaeology
☒	☐	Animals and other organisms
☒	☐	Human research participants
☒	☐	Clinical data
☒	☐	Dual use research of concern

Methods

n/a	Involved in the study	
☒	☐	ChIP-seq
☒	☐	Flow cytometry
☒	☐	MRI-based neuroimaging

Antibodies

Antibodies used	Anti-METTL3 (Invitrogen, #15073-1-AP), anti-m6A (D9D9W)(Cell Signal and Technology, #56593), HA Tag Monoclonal Antibody (2-2.2.14) (Invitrogen, #26183), Flag Tag Monoclonal Antibody (M2) (Sigma, #F1804), Alexa Fluor Plus 488 (Thermo Fisher Scientific, #32723), Anti-FOXM1 Antibody (A-11): (Santa Cruz, #sc-271746, 1:500 dilution; Anti-Sox-2 Antibody (E-4) (Santa Cruz: sc-365823) 1:500 dilution; Anti-Gapdh Antibody GAPDH (D16H11) XP® Rabbit mAb (Cell Signal and Technology, #5174), Vinculin (E1E9V) XP® Rabbit mAb (Cell Signal and Technology, #13901), 1:1000 dilution.
Validation	Antibodies were validated by the vendors and corresponding can be found on the vendor's website.

Eukaryotic cell lines

Policy information about cell lines

Cell line source(s)	HEK293T cells and HeLa cells were purchased from the American Type Culture Collection (ATCC).
Authentication	No method of cell line authentication was used.
Mycoplasma contamination	Cell lines were tested for mycoplasma, but no mycoplasma contamination was detected.
Commonly misidentified lines (See ICLAC register)	No commonly misidentified cell lines were used.
